# Non-invasive CMR-Based Quantification of Myocardial Power and Efficiency Under Stress and Ischemic Conditions in Landrace Pigs

**DOI:** 10.3389/fcvm.2021.689255

**Published:** 2021-07-26

**Authors:** Alessandro Faragli, Alessio Alogna, Chong Bin Lee, Miry Zhu, Niky Ghorbani, Francesco Paolo Lo Muzio, Bernhard Schnackenburg, Christian Stehning, Titus Kuehne, Heiner Post, Leonid Goubergrits, Eike Nagel, Burkert Pieske, Sebastian Kelle, Marcus Kelm

**Affiliations:** ^1^Department of Internal Medicine and Cardiology, Deutsches Herzzentrum Berlin, Berlin, Germany; ^2^Charité – Universitätsmedizin Berlin, Department of Internal Medicine and Cardiology, Campus Virchow-Klinikum, Berlin, Germany; ^3^Berlin Institute of Health, Berlin, Germany; ^4^DZHK (German Centre for Cardiovascular Research), Partner Site Berlin, Berlin, Germany; ^5^Institute for Computational and Imaging Science in Cardiovascular Medicine, Charité – Universitätsmedizin Berlin, Berlin, Germany; ^6^Department of Surgery, Dentistry, Paediatrics and Gynaecology, University of Verona, Verona, Italy; ^7^Department of Medicine and Surgery, University of Parma, Parma, Italy; ^8^Clinical Science, Philips Healthcare, Hamburg, Germany; ^9^Department of Congenital Heart Disease, Deutsches Herzzentrum Berlin, Berlin, Germany; ^10^Department of Cardiology, Contilia Heart and Vessel Centre, St. Marien-Hospital Mülheim, Mülheim, Germany; ^11^Einstein Center Digital Future, Berlin, Germany; ^12^Institute of Experimental and Translational Cardiac Imaging, DZHK Centre for Cardiovascular Imaging, Goethe University Hospital Frankfurt, Frankfurt, Germany

**Keywords:** myocardial efficiency, myocardial power, landrace pigs, dobutamine stress, myocardial ischaemia

## Abstract

**Background:** Myocardial efficiency should be maintained stable under light-to-moderate stress conditions, but ischemia puts the myocardium at risk for impaired functionality. Additionally, the measurement of such efficiency typically requires invasive heart catheterization and exposure to ionizing radiation. In this work, we aimed to non-invasively assess myocardial power and the resulting efficiency during pharmacological stress testing and ischemia induction.

**Methods:** In a cohort of *n* = 10 healthy Landrace pigs, dobutamine stress testing was performed, followed by verapamil-induced ischemia alongside cardiac magnetic resonance (CMR) imaging. External myocardial power, internal myocardial power, and myocardial efficiency were assessed non-invasively using geometrical and functional parameters from CMR volumetric as well as blood flow and pressure measurements.

**Results:** External myocardial power significantly increased under dobutamine stress [2.3 (1.6–3.1) W/m^2^ vs. 1.3 (1.1–1.6) W/m^2^, *p* = 0.005] and significantly decreased under verapamil-induced ischemia [0.8 (0.5–0.9) W/m^2^, *p* = 0.005]. Internal myocardial power [baseline: 5.9 (4.6–8.5) W/m^2^] was not affected by dobutamine [7.5 (6.9–9.0) W/m^2^, *p* = 0.241] nor verapamil [5.8 (4.7–8.8) W/m^2^, *p* = 0.878]. Myocardial efficiency did not change from baseline to dobutamine [21% (15–27) vs. 31% (20–44), *p* = 0.059] but decreased significantly during verapamil-induced ischemia [10% (8–13), *p* = 0.005].

**Conclusion:** In healthy Landrace pigs, dobutamine stress increased external myocardial power, whereas myocardial efficiency was maintained stable. On the contrary, verapamil-induced ischemia substantially decreased external myocardial power and myocardial efficiency. Non-invasive CMR was able to quantify these efficiency losses and might be useful for future clinical studies evaluating the effects of therapeutic interventions on myocardial energetics.

## Introduction

Myocardial efficiency represents the ratio between the external mechanical power required to maintain blood flow against systemic vascular resistance and the contraction power required by the left ventricular (LV) myocardium (1, 2). This mechanical efficiency proportion has increasingly become the focus of research, as it is associated to myocardial performance and myocardial remodeling (3, 4) in patients with cardiac diseases such as valvular heart disease (5, 6), heart failure (7), and cardiomyopathy (8).

Changes in myocardial efficiency can be triggered and altered by several factors, such as medical treatment (9) or surgical intervention (6), inducing hemodynamic changes in the cardiovascular system. Furthermore, efficiency can be influenced by stress and ischemic hemodynamic conditions, which can be pharmacologically induced by dobutamine (10–12) and verapamil (13, 14), respectively. The quantification of myocardial efficiency in such conditions holds the potential for providing insights into myocardial physiology as well as tolerance for stress and ischemia.

Nevertheless, even in resting conditions, the quantification of myocardial efficiency has not been established as a clinical standard since it is traditionally based on invasive measurements (3, 4). More recent concepts for its quantification have suggested the use of positron emission tomography (PET) in combination with concepts of mechanical external power (5, 6). However, PET is limited to specific clinical indications and includes exposure to ionizing radiation. A novel cardiac magnetic resonance (CMR)-based approach has been introduced to assess alterations in mechanical myocardial power and myocardial efficiency (15). The aim of this animal study was to quantify myocardial power and efficiency under stress and ischemic conditions using this non-invasive CMR approach.

## Methods

Data from *n* = 10 (51 ± 10 kg) Landrace pigs were selected from an already published study cohort from our group (16, 17), where dobutamine stress testing and verapamil ischemia induction were performed. Female Landrace swine were fasted overnight with free access to water and then sedated and intubated on the day of the experiment. Anesthesia was continued with fentanyl, midazolam, ketamine, and pancuronium as needed. The anesthesia regimen included a low-dose isoflurane to obtain deeper sedation and stabilize the hemodynamics without impacting much on systemic vascular resistance. Dobutamine infusion was titrated, aiming at a 25% heart rate (HR) increase compared to baseline values, while verapamil was given as a single 2.5-mg bolus, aiming at a 25% decrease of cardiac index (CI). The experimental protocols were approved by the local bioethics committee of Berlin, Germany (G0138/17), and conform to the “European Convention for the Protection of Vertebrate Animals Used for Experimental and Other Scientific Purposes” (Council of Europe No. 123, Strasbourg 1985). The corresponding author had full access to all the data in the study and takes responsibility for its integrity and the data analysis.

### Experimental Protocol

The experimental protocol has been already described in two published papers from our group (16, 17). Briefly, female Landrace pigs (*n* = 10, weight = 51 ± 10 kg) were acutely instrumented, and the animals were transported to the MRI facility for measurements. The pigs were ventilated with an MRI-compatible machine (Titus, Dräger Medical, Germany). After the baseline measurements, two steps were performed as follows: (I) dobutamine-induced stress (Dob) and (II) verapamil-induced ischemia (Ver). Dobutamine infusion was titrated, aiming at a 25% heart rate increase compared to the baseline values, while verapamil was given at boli of 2.5 mg each, aiming at a 25% decrease of CI. At each protocol, MRI images were acquired at short axis (SAX), 2Ch, 3Ch, and 4Ch views. After the MRI measurements, the animals were transported back to the operating room for sacrifice.

### Cardiac Magnetic Resonance

All CMR images were acquired in a supine position using a 3T (Achieva, Philips Healthcare, Best, The Netherlands) MRI scanner with a flexible anterior and a built-in posterior receive coil, where up to 30 coil elements were employed depending on the respective anatomy. All animals were scanned using identical comprehensive imaging protocols. The study protocol included initial scouts to determine the cardiac imaging planes. Cine images were acquired using an ECG-gated balanced steady-state free precession sequence in three LV long-axis (2Ch, 3Ch, and 4Ch) planes. The ventricular two- and four-chamber planes were used to plan stack of SAX slices covering the entire LV. The following imaging parameters were used: repetition time (TR) = 2.9 ms, echo time (TE) = 1.45 ms, flip angle = 45°, acquired in-plane voxel size = 1.9 × 1.9 mm^2^, reconstructed voxel size = 1.0 x 1.0 mm^2^, slice thickness 8 mm, 40 reconstructed cardiac phases, and number of averages (NSA) 2. Flow was quantified using two-dimensional phase-contrast MRI: TR 3.9, ms; TE, 2.4 ms; flip angle, 15°; 30 reconstructed cardiac phases; acquired in-plane voxel size, 2.1 × 2.1 mm^2^; reconstructed voxel size, 1.5 × 1.5 mm^2^; slice thickness, 10 mm; velocity encoding, 200 cm/s; and NSA, 2. The sequences were obtained (a) perpendicular to the aorta to assess peak flow and aortic valve regurgitation as well as (b) between the mitral valve inflow and the left ventricular outflow tract to quantify the auxobaric contraction time, the isovolumetric contraction time, and the aortic pressure gradient, respectively. All images were acquired during normal respiration with no respiratory motion correction, as respiration-induced bulk cardiac motion was absent or very small in these animals. All images were analyzed offline using Medis Suite (version 3.1, Leiden, The Netherlands) and View Forum (R6.3V1L7 SP1, Philips Medical Systems Nederland B.V) in accordance to the consensus for quantification function and flow using CMR.

### Myocardial Power and Power Efficiency

**Figure 1:**
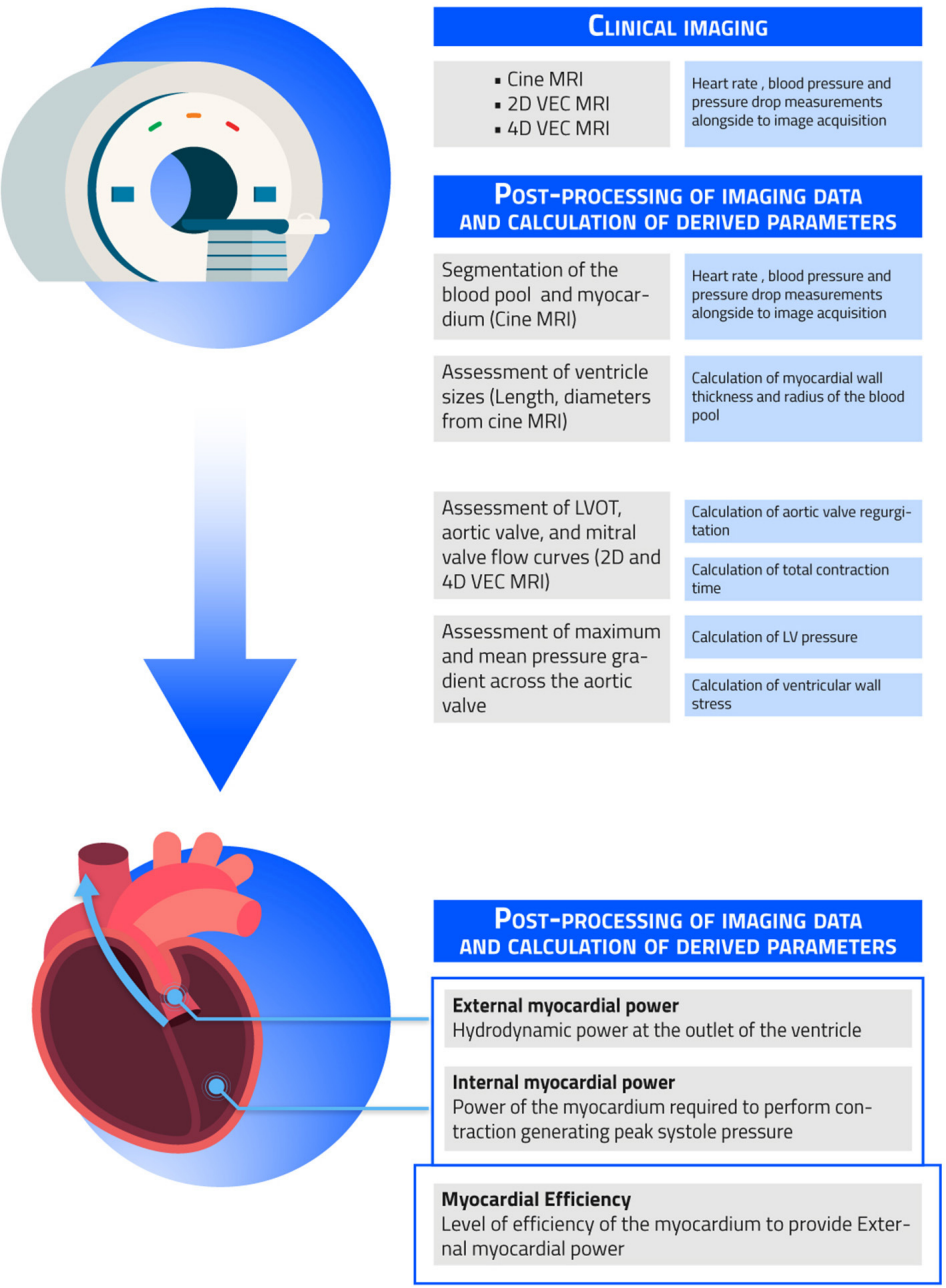
Clinical imaging and post-processing workflow. Shown in the figure are the necessary steps needed to obtain the non-invasively measured external myocardial power, internal myocardial power, and myocardial efficiency.

The clinical imaging and post-processing workflow to obtain CMR-derived myocardial power and efficiency is described in Figure 1.

Power is the rate of transferring or converting energy per unit time. Cardiac power output (CPO) defines the power needed to pump blood against a given afterload of the systemic circulation.

$$\begin{array}{l} CPO\;\left(W\right)=\frac{MAP\;*\;CO_{total}}{451}\; \end{array}$$

with MAP = mean arterial pressure (converted from mmHg to Pa) measured invasively, 451 = the unit conversion in watt, and CO_total_ = total cardiac output (converted from L/min to qm/s). Cardiac output (CO) was calculated by the Simpson method to measure LV volumes using SAX MRI images. Simpson's technique is based on Simpson's rules, which for our purposes were essentially the sum of the cross-sectional areas of each slice accounting for slice thickness and the interval between slices in a stack of contiguous slices covering the entire LV. LV contours in all slices required to measure the cross-sectional areas in each slice were assessed manually. External myocardial power (EMP) defines the power needed to pump blood against a given afterload at the LVOT.

$$\begin{array}{l} EMP\;\left(W\right)\;=\left(MAP+mean{△P}_{AV}\right)\;*\;CO_{total}\; \end{array}$$

The *mean△P*_*AV*_ is the mean pressure gradient across the aortic valve. In our cohort, EMP is nearly equivalent to the concept of cardiac power output and circulatory power (15), given the absence of aortic valve stenosis and aortic valve regurgitation.

The power required by the LV myocardium to perform contraction during systole is defined as internal myocardial power (IMP) (15, 18):

$$\begin{array}{l} IMP\;\left(W\right)\;=\frac{V_{wall}\;*\;\sigma_{wall}}{t_{CS}}\; \end{array}$$

where *V*_*wall*_ = myocardial wall volume, σ_*wall*_ = peak systolic wall stress, and *t*_*CS*_ = left ventricular systolic contraction time. EMP and IMP have been indexed to body surface area (BSA) to allow a better comparison between individuals. Wall stress was calculated using a simplified approach of the law of Laplace:

$$\begin{array}{l} \sigma_{wall}\;=P_{sys}\;*\;\frac{R_{BP}}{2\;*\;S_{wall}} \end{array}$$

Where *P*_SYS_ = LV peak systolic pressure, *R*_BP_ = mean radius of the blood pool, and *S*_wall_ = mean myocardial wall thickness. *S*_wall_ and *R*_BP_ during systole were averaged from LV segmentations, considering LV as a cylindrical geometry for the correction of potential regional differences. *P*_sys_ = sum of the systolic blood pressure measured at the right arm and the maximum pressure gradient across the aortic valve. The ratio between IMP to the resulting EMP (IMP/EMP) is defined as myocardial efficiency.

### Statistical Analysis

Continuous data are expressed as median and interquartile range (Q1–Q3) unless stated otherwise. Data distribution was tested using Shapiro–Wilk and Shapiro–Francia tests. The correlations between the CMR parameters of indexed IMP, indexed EMP, myocardial efficiency, and the hemodynamic parameters of CPO, CI, and left ventricular ejection fraction (LVEF) were assessed by linear regression analysis.

Wilcoxon signed rank test was used to assess differences between baseline and the intervention. The significance level was set at 0.05. Data were analyzed using Stata 15.1 (StataCorp, College Station, TX, USA) and GraphPad 8.0.2 (GraphPad Software, Inc., La Jolla, CA, USA).

## Results

Power and efficiency analyses were performed in *n* = 10 pigs. The hemodynamic and the CMR-based geometrical and functional parameters of the pigs at rest, under dobutamine and under verapamil, are reported in Table 1. The following hemodynamic data have been previously published (17), where a significant increase of HR, mean aortic pressure, and CPO under Dob and a respective significant decrease during Ver were observed; stroke volume and LVEF did not decrease significantly under Dob but decreased significantly instead during Ver.

**Table 1:** Clinical characteristics, cardiac magneic resonance geometric and functional parameters, and invasively measured hemodynamic parameters of pigs at rest, under stress (dobutamine), and under ischemia (verapamil).

**Subjects (*n* = 10)**	**BL**	**Dob**	**Ver**	***p*-value** **(BL vs. Dob)**	***p*-value** **(BL vs. Ver)**
sBP (mmHg)	108 (101–117)	136 (127–147)	87 (78–106)	0.007	0.005
dBP (mmHg)	79 (60–87)	84 (57–97)	58 (50–66)	0.646	0.012
Myocardial mass/BSA (g/m^2^)	41 (34–52)	41 (34–52)	41 (34–52)	–	–
Myocardial volume (ml)	38 (32–50)	38 (32–50)	38 (32–50)	–	–
SV_flow_ (ml)	57 (44–80)	52 (45–78)	42 (47–53)	0.386	0.005
CO_flow_ (L/min)	5.6 (4.8–6.5)	6.0 (4.6–8.0)	3.9 (3.1–4.9)	0.220	0.013
Distensibility aorta ascendens (10^−3^ mmHg^−1^)	11.2 (7.7–14.8)	9.7 (6.1–14.6)	5.5 (4.2–7.7)	0.721	0.017
Distensibility aorta descendens (10^−3^mmHg^−1^)	7.0 (4.4–9.6)	6.7 (4.5–8.6)	4.9 (1.5–7.0)	0.721	0.139
External myocardial power (W/m^2^)	1.3 (1.1–1.6)	2.3 (1.6–3.1)	0.8 (0.5–0.9)	0.005	0.005
Internal myocardial power (W/m^2^)	5.9 (4.6–8.5)	7.5 (6.9–9.0)	5.8 (4.7–8.8)	0.241	0.878
Myocardial efficiency (%)	21 (15–27)	31 (20–44)	10 (8–13)	0.059	0.005

*BSA, body surface area; BL, baseline; Dob, dobutamine infusion; Ver, verapamil infusion; sBP, systolic blood pressure; dBP, diastolic blood pressure; mAOP, mean arterial pressure; EDV, end-diastolic volume; ESV, end-systolic volume; LVEF, left ventricular ejection fraction; HR, heart rate; SV, stroke volume; CO, cardiac output*.

*Data are presented as median and interquartile range*.

### Hemodynamic Parameters

Relevant differences for systolic blood pressure were seen between pigs under stress, whereas no differences were seen for diastolic blood pressure and mean arterial pressure between interventions (stress and ischemia) and rest. After stress induction with dobutamine, the heart rate increased significantly, while HR did not change after ischemia induction with verapamil.

CO, calculated by the Simpson method, significantly increased during stress with dobutamine and also significantly decreased vs. baseline during ischemia. Invasively measured CPO significantly increased during dobutamine stress, and a significant decrease was observed during ischemia with verapamil. LVEF increased non-significantly during dobutamine but significantly decreased during verapamil.

### Heart Power Analysis

**Figure 2:**
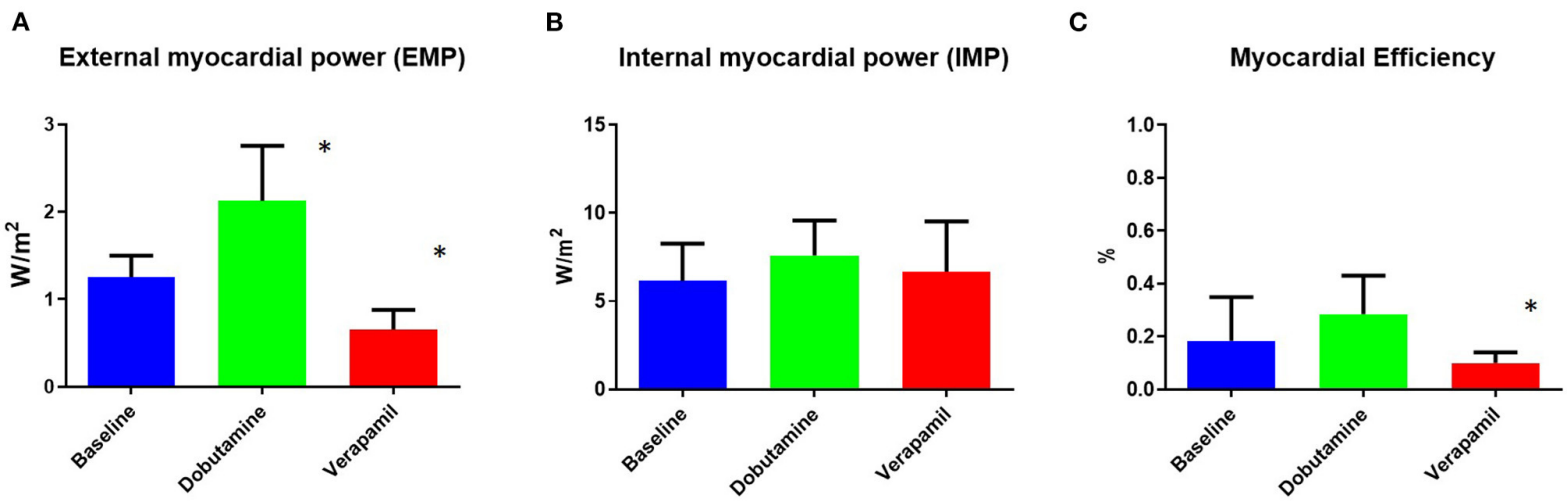
Non-invasively measured myocardial power and efficiency at baseline and at dobutamine and verapamil states. Body surface area (BSA)-indexed external myocardial power **(A)** was found to be significantly different during both dobutamine-induced stress and verapamil-induced ischemia. No difference between states was found for BSA-indexed internal myocardial power **(B)**. Myocardial efficiency **(C)** was found to be significantly different only during verapamil-induced ischemia. EMP, external myocardial power; IMP, internal myocardial power. *p < 0.05 vs. baseline.

EMP, IMP, and myocardial efficiency were assessed in all groups using CMR. EMP changed significantly during both dobutamine-induced stress and also after ischemia induction (Figure 2A). There were instead no significant differences in IMP after stress with dobutamine or ischemia with verapamil (Figure 2B). Myocardial efficiency changed significantly from baseline to verapamil, but only a decreasing trend was observed between baseline and dobutamine (Figure 2C).

### Comparison With Hemodynamic Parameters (Left Ventricular Ejection Fraction, Cardiac Index, and Cardiac Power Output)

**Figure 3:**
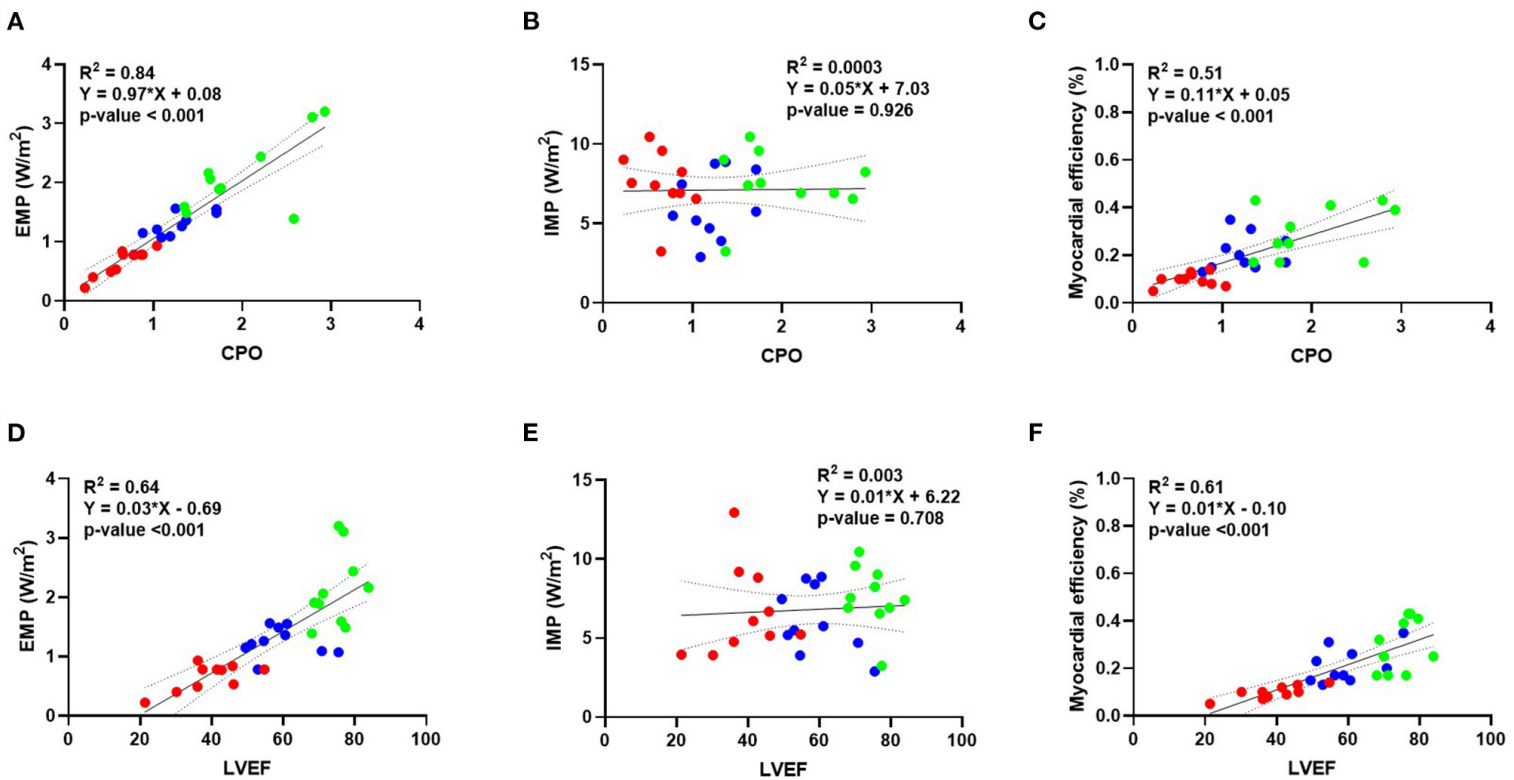
Correlation between external myocardial power, internal myocardial power, and myocardial efficiency vs. different invasive parameters of hemodynamics. Linear regression analysis showing a good correlation between external myocardial power vs. CPO **(A)** and LVEF **(D)**. No correlation is instead observed for internal myocardial power and CPO **(B)** or LVEF **(E)**. A moderate correlation is observed for myocardial efficiency vs. CPO **(C)** and LVEF **(F)**. Blue dots represent baseline, green dots represent dobutamine, and red dots represent verapamil. BSA, body surface area; CPO, cardiac power output; LVEF, left ventricular ejection fraction.

The linear regression analysis showed a good correlation between EMP and CPO (Figure 3A) and between external myocardial power and LVEF (Figure 3D). No correlation was observed between IMP and CPO (Figure 3B) and vs. LVEF (Figure 3E). A moderate correlation was observed instead between myocardial efficiency and CPO (Figure 3C) and LVEF (Figure 3F).

## Discussion

### Summary of Main Findings

The results of our non-invasive MRI approach show that myocardial efficiency was not altered during dobutamine stress testing, whereas efficiency was decreased during verapamil-induced ischemia. While external myocardial power directly reflects the resulting hemodynamic responses under both stress and ischemic conditions, myocardial efficiency reveals the coupling between the left ventricle and the circulatory system.

### External Myocardial Power

External power has been shown to predict mortality in patients with cardiogenic shock or outcome in chronic heart failure patients better than LVEF (19, 20). In a study from our group, we were able to show that CPO, as an external power measure, accurately reflects left ventricular stroke work per minute over a wide range of inotropic states (21). The area of the pressure–volume loop reflecting the external work of the heart is also called left ventricular stroke work (LV SW). Through an invasive pressure–volume analysis, this represents the most comprehensive way to describe ventricular performance (22). While LVEF provides a load-dependent measurement of heart function, LV SW and, in turn, CPO offer load-independent information extremely relevant in patients with altered inotropy due to heart failure (21).

### Internal Myocardial Power and Myocardial Efficiency

The energy that the myocardium needs to generate external power and the resulting efficiency have been of particular interest from a clinical and a research standpoint, respectively. In the diseased myocardium, this efficiency is known to be reduced (3), and it has long been discussed whether this energy loss can help to describe the pathophysiology in the diseased state (8). In line with these concepts, Güçlü et al. (6) have demonstrated that myocardial efficiency plays a crucial role in myocardial remodeling processes in aortic valve stenosis and improvements can be measured after surgical valve replacement. These changes in external myocardial efficiency were shown to correlate to the changes in myocardial oxygen consumption (VO_2_). A surrogate for the mechanical internal power that does not require exposure to ionizing radiation and that can be related to the resulting external power (external myocardial efficiency) has been recently introduced (15). Similar to the findings by Güçlü et al. (6), internal myocardial power was found to be increased with a resulting reduced efficiency in patients with aortic valve stenosis compared to controls as what would be expected under conditions of increased afterload. While external myocardial power was not different between aortic stenosis patients and controls as long as the EF was preserved, circulatory efficiency was found below control levels, even in patients with normal EF (15).

Within this animal cohort, when the myocardium was not impaired, its efficiency was found to be at human control levels under resting conditions (15). During a dobutamine-induced stress response, the efficiency did not change. Based on previous concepts of myocardial energy (3), losses would not be expected under healthy conditions. In line with these concepts, an impaired myocardial efficiency was found under ischemic conditions when the LV contraction power resulted in less external myocardial power. The internal myocardial power already includes aspects of cardiac morphology, and future research is required to assess whether myocardial fibrosis and histological changes will be reflected in power measurements (23). Note that IMP did not changed significantly between the three conditions. We suppose that this is due to a healthy heart, which is able to cover drug-simulated changes in afterload (stress and ischemia). A decrease in IMP means better heart work efficiency that we cannot expect in a healthy heart. An increase in IMP means lower efficiency in heart work, which should be associated with pathological changes of the heart which, however, require time. Short-time changes in heart work conditions, as investigated in the current study, cannot be associated with such a pathological change. In a recently published work which investigated myocardial power and efficiency before and after treatment of the aortic valve, a significant decrease in IMP and a respective increase in efficiency due to treatment of the aortic valve were shown (24). Hence, IMP is not a constant value.

### Invasively Measured CPO Against External Myocardial Power

In our study, we were able to show that external myocardial power measured with CMR is a feasible technique able to correlate CPO, measured invasively. This represents an advancement in CMR-based assessment of heart function, adding a potential new parameter that can be utilized in patients with compromised LV function.

### Limitations

The calculation of myocardial power and efficiency focuses on systole as it accounts for most of the energy expenditure of the heart. Myocardial energetics were not considered in diastole even though diastolic relaxation is an active energy-requiring process involving ATP and oxygen consumption. Diastolic dysfunction plays a major role in the pathophysiology of several cardiac diseases such as heart failure, but the role of myocardial efficiency regarding its pathophysiology is still unknown. Hence, novel concepts are required to quantify myocardial efficiency in the diastolic phase of the heart to get a deeper understanding of the pathophysiology of diastolic heart failure. Our approach did not include metabolic measures of myocardial energy consumption which traditionally had to be assessed with the use of an invasive conductance catheter. Modern concepts use PET, which allows measuring of myocardial energy consumption by indirectly quantifying metabolic oxygen consumption from the coronaries with the disadvantage of using ionizing radiation. This study was a pure mechanical MRI-only approach calculating the internal myocardial power with the assumption that internal power is the mechanical potential power generated by myocardial contraction. Furthermore, LV wall stress was calculated using a simplified approach of the law of Laplace. The geometrical shape of the LV as well as regional strain both determine LV wall stress and, subsequently, impact internal myocardial power. Hence, more precise models should be applied to calculate internal myocardial power more accurately in future projects. The current study was limited to testing of dobutamine and verapamil drugs. The effect of other drugs such as dypiridamole, adenosine, or levosimendan should be investigated in frames of future studies. This study was an animal study with a small cohort of *n* = 10. As animal studies can be an imprecise predictor of the reactions and physiology of humans, future clinical trials need to be carried out to prove our results in patients under various disease states.

## Conclusions

This study underlines the concept of assessing internal and external myocardial power and their resulting efficiency in a completely non-invasive CMR-based approach. Efficiency measures were shown to maintain stability during stress testing as long as the myocardium is not impaired. Under ischemic conditions, however, quantifiable efficiency losses occurred. This study underlines the promising potential of the non-invasive approach in human subjects, in particular in those with altered myocardial performance.

## Data Availability Statement

The raw data supporting the conclusions of this article will be made available by the authors, without undue reservation.

## Ethics Statement

The experimental protocols were approved by the local bioethics committee of Berlin, Germany (G0138/17), and conform to the European Convention for the Protection of Vertebrate Animals used for Experimental and other Scientific Purposes (Council of Europe No 123, Strasbourg 1985).

## Author's Note

Professor Dragun passed away on December 28, 2020. This publication is dedicated to her memory as a mentor, role model, and stellar scientist.

## Author Contributions

SK, HP, AA, and MK: conceptualization and methodology. LG: software. MK, AF, and LG: formal analysis. CL, MZ, NG, MK, and AF: writing—original draft preparation. AF, AA, CL, MZ, NG, FL, BS, CS, TK, HP, LG, EN, BP, SK, and MK: writing—review and editing. All authors contributed to the article and approved the submitted version.

## Conflict of Interest

BP reports having received consultancy and lecture honoraria from Bayer Daiichi Sankyo, MSD, Novartis, Sanofi-Aventis, Stealth Peptides, and Vifor Pharma and editor honoraria from the *Journal of the American College of Cardiology*. SK reports receiving grants from Philips Healthcare and speaker honoraria from Medis. CS and BS are employees of Philips Healthcare. The remaining authors declare that the research was conducted in the absence of any commercial or financial relationships that could be construed as a potential conflict of interest.

## Publisher's Note

All claims expressed in this article are solely those of the authors and do not necessarily represent those of their affiliated organizations, or those of the publisher, the editors and the reviewers. Any product that may be evaluated in this article, or claim that may be made by its manufacturer, is not guaranteed or endorsed by the publisher.
